# Comprehensive assessment of amino acid substitutions in the trimeric RNA polymerase complex of influenza A virus detected in clinical trials of baloxavir marboxil

**DOI:** 10.1111/irv.12821

**Published:** 2020-10-24

**Authors:** Takashi Hashimoto, Keiko Baba, Kae Inoue, Miyako Okane, Satoshi Hata, Takao Shishido, Akira Naito, Steffen Wildum, Shinya Omoto

**Affiliations:** ^1^ Shionogi & Co., Ltd. Osaka Japan; ^2^ Shionogi Techno Advance Research, Co., Ltd. Osaka Japan; ^3^ F. Hoffmann‐La Roche Ltd. Basel Switzerland

**Keywords:** antiviral, baloxavir, influenza virus, replicative capacity, susceptibility

## Abstract

**Background:**

Baloxavir marboxil (BXM) is an approved drug that selectively targets cap‐dependent endonuclease on PA subunit in the RNA polymerase complex of influenza A and B viruses. Amino acid substitutions at position 38 in the PA subunit were identified as a major pathway for reduced susceptibility to baloxavir acid (BXA), the active form of BXM. Additionally, substitutions found at positions E23, A37, and E199 in the PA subunit impact BXA susceptibility by less than 10‐fold.

**Methods:**

We comprehensively evaluated the impact of novel amino acid substitutions identified in PA, PB1, and PB2 subunits in BXM clinical trials and influenza sequence databases by means of drug susceptibility and replicative capacity.

**Results:**

PA/I38N in A(H1N1)pdm09 and PA/I38R in A(H3N2) were newly identified as treatment‐emergent substitutions in the CAPSTONE‐2 study. The I38N substitution conferred reduced susceptibility by 24‐fold, whereas replicative capacity of the I38N‐substituted virus was impaired compared with the wild‐type. The I38R‐substituted virus was not viable in cell culture. All other mutations assessed in this extensive study did not significantly affect BXA susceptibility (< 2.4‐fold change).

**Conclusion:**

These results provide additional information on the impact of amino acid substitutions in the trimeric viral polymerase complex to BXA susceptibility and will further support influenza surveillance.

## 1 INTRODUCTION

Influenza is an acute infectious disease caused by the influenza virus, and the worldwide epidemics each year result in approximately 3‐5 million seriously ill cases and approximately 290 000‐650 000 deaths.^1^ Antiviral treatment is recommended for the management of influenza infections, particularly in high‐risk individuals such as elderly and immunocompromised persons. Neuraminidase inhibitors (NAIs: oseltamivir, zanamivir, peramivir) are widely used as the current treatment for influenza,^2^ while adamantanes, M2 ion channel inhibitors, are no longer used due to widespread resistance in circulating influenza viruses.^3^, ^4^ While NAI‐resistant A(H1N1)pdm09 viruses are currently only detected at a frequency of <1% among circulating viruses,^2^ community clusters of such variant viruses have been detected,^5^, ^6^, ^7^ emphasizing the need for antivirals with a novel mechanism of action.

Baloxavir marboxil (BXM) became available for the treatment of uncomplicated influenza in otherwise healthy and high‐risk patients in a number of countries, following its approval in Japan and the United States in 2018.^8^, ^9^ Baloxavir acid (BXA), the active form of BXM, selectively and potently blocks a catalytic center of cap‐dependent endonuclease (CEN) located in the polymerase acid (PA) protein of the influenza polymerase complex, which consists of PA, polymerase basic 1 (PB1), and PB2 subunits.^10^, ^11^ The CEN is highly conserved across all types of influenza viruses^12^ and plays an essential role in the transcription, protein synthesis, and viral genome replication,^13^ and therefore, BXA displays broad‐spectrum activity against influenza A, B, C, and D viruses.^14^, ^15^ In clinical trials, single‐dose BXM treatment was superior to placebo in relieving influenza symptoms and, additionally, superior to both oseltamivir and placebo in reducing the viral load.^8^, ^9^ However, amino acid (AA) substitutions at position I38 (T/M/F) in the PA subunit have been identified as the most common treatment‐emergent substitutions associated with reduced susceptibility to BXA.^11^, ^16^ Influenza surveillance studies conducted in Japan during the 2018‐19 influenza season confirmed treatment‐emergence of PA/I38T and PA/I38M variants in A(H3N2)‐infected subjects.^17^, ^18^ A(H1N1)pdm09 and A(H3N2) viruses harboring PA/I38T substitution were detected in some few subjects without prior BXM treatment, suggesting the possibility of human‐to‐human transmission of the variant viruses.^18^, ^19^ In addition to the I38 substitutions, E23K/G, A37T, and E199G substitutions were identified in the PA subunit that affect BXA susceptibility by less than 10‐fold.^11^, ^20^, ^21^ Therefore, consecutive monitoring of variant viruses with reduced BXA susceptibility is required to identify new potential genetic markers for the purpose of influenza surveillance.

It has been well demonstrated that mutations in NA conferring resistance to NAIs can negatively impact the viral replicative capacity, but additional HA mutations can also compensate these fitness cost.^22^ K229R in the PB1 subunit of influenza A viruses confers resistance to the viral RNA polymerase inhibitor favipiravir, and the fitness cost caused by this mutation can be compensated by a P653L substitution in PA that restores the fitness while maintaining favipiravir resistance.^23^ Therefore, AA substitutions located at distal position from drug‐binding sites may impact drug sensitivity or compensate impaired fitness.

Here, we report phenotypic analyses of AA substitutions in PA, PB1, and PB2 subunits, which were detected in clinical trials and influenza surveillance. This additional information on BXA susceptibility and replicative capacity of viruses with these substitutions will further support influenza surveillance.

## 2 MATERIALS AND METHODS

### 2.1 Clinical trials

A multicenter, randomized, double‐blind, controlled phase 2 study was conducted during the 2015‐16 influenza season with BXM in Japanese adults aged 20‐64 years with uncomplicated influenza (Japic CTI‐153090).^24^ In the subsequent 2016‐17 influenza season, an open‐label study was conducted with BXM in otherwise healthy pediatric patients aged 6 months to <12 years with uncomplicated influenza (Japic CTI‐163417).^25^ The CAPSTONE‐1 study (ClinicalTrials.gov NCT02954354) was conducted in the United States and Japan as a double‐blind, placebo‐ and oseltamivir‐controlled, randomized trial that enrolled outpatients aged 12 to 64 years with influenza‐like illness in 2016‐17.^8^ The CAPSTONE‐2 study (ClinicalTrials.gov NCT02949011) was a double‐blind, placebo‐, and oseltamivir‐controlled trial involving outpatients aged ≥12 years in 551 sites in 17 countries and territories, and eligible patients had clinically diagnosed influenza‐like illness, at least one risk factor for influenza‐related complications (eg, age >65 years), and a symptom duration of ≤48 hours.^9^ Written informed consent was obtained from all the patients in clinical trials, and all methods related to clinical samples were derived according to standard operating procedures in accordance with the protocol approved by the institutional review board (IRB), all applicable regulatory requirements, and the current Good Clinical Practice (GCP) guidelines.

### 2.2 Compounds, cells, and viruses

Baloxavir acid (S‐033447; BXA) was synthesized at Shionogi & Co., Ltd., and favipiravir was purchased from PharmaBlock Sciences, Inc., and AdooQ BioScience. MDCK, RPMI2650, and 293T cells were cultured as described previously.^11^ For generation of recombinant viruses by reverse genetics, the plasmid set of rgA/WSN/33 (H1N1), rgA/Victoria/3/75 (H3N2), and rgB/Maryland/1/59 were used as described previously.^11^

### 2.3 Genotypic analyses of clinical samples

In the clinical trials, Sanger sequencing was conducted using paired pre‐ and post‐treatment nasopharyngeal/pharyngeal swab samples from BXM‐treated subjects to identify treatment‐emergent AA substitutions. In the CAPSTONE‐2 study, next‐generation sequencing (NGS) was also employed with the samples meeting the following criteria: (a) subjects shedding A(H3N2) viruses with PA/I38T substitution, (b) virology data (viral titer and RNA) are available at days 1, 2, 3 or 4, and 5 or 6, and (c) virus rebound was detected, defined as >1.5 log_10_TCID_50_/mL increases in viral titer from previous adjacent time point. NGS was conducted as reported previously, and a threshold frequency of >1% was adopted for calling variant viruses.^16^

### 2.4 Phenotypic analyses of variant viruses

The plaque reduction assay was conducted as described previously.^11^ A series of mutant influenza viruses was generated by Virapur (San Diego, CA, USA) using reverse genetics to determine drug sensitivity to BXA using Virapur's ViraDot Assay. The assay is a modification of the HINT assay developed by Gubareva et al^20^ and is based on a single round of replication of influenza virus in MDCK cells. Briefly, 3 × 10^4^ MDCK cells/well were plated in 96‐well plates 1 day prior to infection. Cells were infected (500 Dot‐forming units/well), and BXA serial dilutions were added. Plates were incubated overnight (22 ± 2 hours) at 37°C (A viruses) or 34°C (B virus) before the cells were fixed and permeabilized with ice‐cold 100% methanol. Cells were probed with a mouse monoclonal anti‐A/NP antibody (Millipore Sigma MAB8251) against influenza A and anti‐B/NP (Millipore Sigma MAB8661) against influenza B for 1 hour at 37°C and washed three times with PBS before anti‐mouse IgG peroxidase‐labeled secondary polyclonal antibody (Sigma #A3682) was added and incubated at 37°C for 1 hour. Cells were washed three times with DPBS, and virus‐infected cells were detected using TrueBlue substrate (KPL, Cat# 5510‐0050/‐0030) and the CTL ImmunoSpot System with the BioSpot software module BioSpot 7.0.23.2 Professional. EC_50_ values were determined from dose‐response curves using GraphPad Prism.

Evaluation of virus‐replicative capacity was previously described.^11^ Briefly, 2 × 10^5^ cells/well MDCK or 1 × 10^6^ cells/well RPMI2650 cells were seeded on 24‐well plates 1 day prior to infection. MDCK and RPMI2650 cells were infected with 10 and 100 TCID_50_/well of the viruses, respectively. The infected cells were incubated at 37°C in a 5% CO_2_ incubator for 1 hour, followed by exchanging the inoculum to MEM containing 3 μg/mL trypsin and incubation at 37°C in the 5% CO_2_ incubator. The culture supernatants were collected at the indicated time points, and viral titers (log_10_TCID_50_/mL) were determined on MDCK cells.

## 3 RESULTS

### 3.1 Assessment of novel PA/I38X substitutions detected in clinical trials

Resistance monitoring in phase 2 (T0821) and pediatric (T0822 [Japic CTI‐163417]) trials revealed treatment‐emergent I38T/F/M substitutions in PA, which confer reduced susceptibility to BXA (Table 1 and Table S1).^11^ In order to assess treatment‐emergent AA substitutions associated with reduced susceptibility to BXA in phase 3 (T0831, CAPSTONE‐1 [NCT02954354] and T0832, CAPSTONE‐2 [NCT02949011]), Sanger sequencing was conducted with paired pre‐ and post‐treatment swab samples from BXM‐treated subjects. While in the CAPSTONE‐1 study, only I38T/M substitutions were detected, in the CAPSTONE‐2 study, PA/I38N‐substituted A(H1N1)pdm09 viruses were newly identified from one BXM‐treated patient. Additionally, PA/I38T‐, I38T/I‐, and I38M‐substituted A(H3N2) viruses were detected from 10, 2, and 1 BXM‐treated subject, respectively, and PA/I38T‐substituted type B viruses were detected from 1 BXM‐treated patient. Moreover, by using NGS analysis with higher sensitivity compared with Sanger sequencing, we found that PA/I38R was temporary detected at day 5 with a frequency of 7.6% in the virus population among one of the 10 subjects with PA/I38T A(H3N2) viruses. Finally, PA/I38S‐substituted A(H1N1)pdm09 viruses and polymorphic PA/I38V and PA/I38L were reported in the literature during 2018/19 influenza season.^19^, ^26^, ^27^

**Table 1:** Susceptibility of the recombinant viruses with PA I38 substitutions to baloxavir acid

Amino acid substitution in PA subunit		FC of BXA	
		rgA/WSN/33 (H1N1)	rgA/Victoria/3/75 (H3N2)
Treatment‐emergent substitutions	I38T	**27.24** ^a^	**56.59** ^a^
	I38F	**10.61** ^a^	20.13^a^
	I38M	13.15^a^	**13.77** ^a^
	I38N	**23.66**	10.32
	I38S	**12.43** ^b^	5.85
	I38R	Not rescued^c^	**Not rescued** ^c^
Polymorphic substitutions	I38V	**2.18**	1.83
	I38L	**6.33** ^b^	2.17

Note

Susceptibility of I38‐substituted viruses to baloxavir acid (BXA) was determined by plaque reduction assay, and fold change (FC) was calculated as relative EC_50_ of each tested virus to that of the cognate wild‐type virus. Bold, clinically confirmed amino acid substitutions.

^a^ Data from reference.^11^

^b^ Data obtained from the current study are shown, while FCs of A(H1N1)pdm09 viruses with I38S and I38L were previously reported as 52‐ and 9‐fold, respectively.^26^

^c^ The recombinant PA/I38R viruses were not reverse‐genetically rescued.

**Figure 1:**
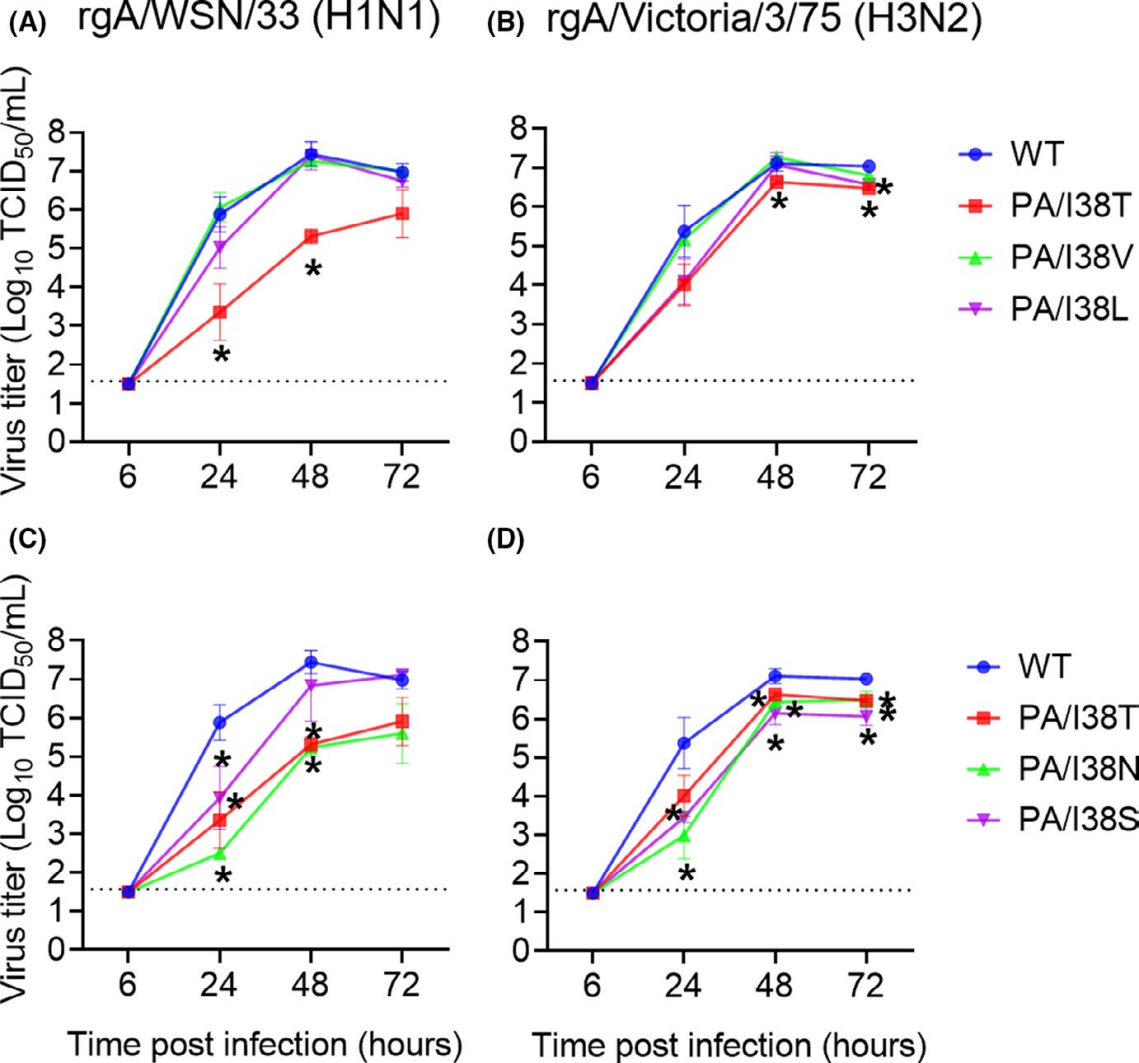
Replicative capacity of variant viruses with indicated PA/I38 substitutions in MDCK cells. MDCK cells were infected with wild‐type (WT) or indicated mutant viruses based on rgA/WSN/33(H1N1) (A, C) and rgA/Victoria/3/75(H3N2) (B, D). The culture supernatants were collected at the indicated time points, and viral titers (TCID_50_/mL) were determined in MDCK cells. Each plot represents the mean and standard deviation of triplicate experiments. The lower limit of quantification of the virus titers was indicated by a dashed line. *P < .05 to WT by Welch's *t* test at the indicated time point

In order to assess the impact of these detected I38 substitutions on BXA susceptibility, the recombinant A(H1N1) and A(H3N2) viruses harboring the individual substitution were generated and subjected to susceptibility testing. Plaque reduction assay revealed that the A(H1N1) viruses with polymorphic I38V and L substitutions displayed reduced BXA susceptibility by 2‐fold and 6‐fold, respectively (Table 1), consistent with a previous report.^26^ The replicative capacities of the A(H1N1) and A(H3N2) viruses with I38V and L substitutions were comparable to those of the wild‐type viruses in canine MDCK and human RPMI2650 cells (Figure 1 and Figure S1). Viruses bearing the I38N and I38S substitutions showed reduced BXA susceptibility by 24‐fold and 12‐fold, respectively (Table 1), but also, the recombinant A(H1N1) and A(H3N2) viruses with I38N and I38S substitutions exhibited significant fitness cost in MDCK and RPMI2650 cells (Figure 1 and Figure S1). The I38R virus could not be obtained by reverse genetics, suggesting I38R conferred severe growth defect to the virus.

### 3.2 Assessment of non‐I38 PA substitutions and PB1/2 substitutions detected in clinical trials

Impact of PA substitutions at other positions than I38 PA on BXA susceptibility was also assessed. Results for AA substitutions identified in T0821 and T0822 clinical trials were previously reported.^11^ None of the newly tested AA substitutions identified in the clinical trials T0831 and T0832 did significantly impact BXA susceptibility (< 3‐fold change in EC_50_) (Table S1).

Treatment‐emergent AA substitutions in PB1 and PB2 subunits were analyzed in T0821, T0822, and T0831 clinical trials. Sanger sequencing was conducted with all paired pre‐ and post‐treatment swab samples from BXM‐treated subjects in studies T0821 and T0822. In study T0831, sequencing of the PB1 and PB2 genes was performed on samples from BXM‐treated patients not exhibiting a treatment‐emergent substitution at position 38 in the PA gene and identified as non‐responders based on the following criteria: (a) virus rebound (virus titer rise of ≥ 0.6 log_10_ TCID_50_/mL between consecutive time points), or (b) continued virus shedding (virus titer > 1.5 log_10_ TCID_50_/mL at day 5 and beyond), or (c) no reduction in virus titer (no change or rise in virus titer between consecutive time points). All detected PB1/2 substitutions were then subjected to susceptibility testing using the plaque reduction assay. None of the tested AA substitutions in PB1/2 did significantly impact BXA susceptibility (EC_50_ fold change ranged from 0.53 to 1.70) (Table S1).

### 3.3 Assessment of PA and PB2 substitutions identified in extended analyses of clinical trial data

Amino acid substitutions at positions associated with baloxavir resistance were identified from NCBI database influenza sequences and from extended analyses of virologic data from clinical trials (Table S2). Substitutions potentially associated with a reduced virologic response (defined as a significantly reduced change from baseline on day 2 in virus titer relative to the virus type/subtype subset distribution), virus rebound, or elevated (≥90 percentile) baseline EC_50_ values of virus isolated from clinical specimens were assessed for their impact on BXA susceptibility using recombinant viruses and the ViraDot assay. None of the identified and tested 21 AA substitutions in PA and PB2 significantly affected BXA susceptibility (< 1.5‐fold change by means of EC_50_ values) (Table S2).

## 4 DISCUSSION

In this study, we characterized PA/I38 substitutions detected in clinical studies (I38T/F/M/N/R/S) and as naturally occurring polymorphisms (I38V/L). PA/I38N was newly identified in the clinical setting, and we demonstrated that BXA susceptibility of I38N viruses was reduced compared with wild‐type virus (24‐fold for A(H1N1) and 10‐fold for A(H3N2)). In addition, the replicative capacity of I38N viruses was reduced to a comparable level to I38T viruses. The genetic barrier to the development of reduced susceptibility is often defined as the number of nucleotide changes required for the AA change. All detected PA/I38 substitutions can develop through a single nucleotide change, suggesting that the introduction of two nucleotide changes may make it difficult for other I38‐substituted viruses to appear.

An arbitrary 3‐fold threshold has been recently used in surveillance screening to define reduced BXA susceptibility.^20^ Although we have comprehensively tested individual AA substitutions in PA, PB1, and PB2 subunits in viral RNA polymerase complex, only known AA positions were detected as substitutions that confer reduced susceptibility by more than 3‐fold change in EC_50_. The body of data supports that I38 substitutions are the major pathway for reduced BXA susceptibility. Rare changes at E23, A37, and E199, found with 0.07% to 0.44% frequency in clinical treatment trials, should be monitored as non‐I38 substitutions. However, the cutoff value at 3‐fold can exceed dependent on robustness of assay systems, and therefore, standardization of susceptibility testing with BXA may be important.

Since AA substitutions in PA protein combined with I38 substitutions may affect functional compensation for fitness cost, we further investigated whether AA substitutions associated with I38 substitutions identified in the clinical trial were compensatory mutations. The replicative capacity was previously evaluated for substitutions A20S + I38F and I38T + E623K,^11^ and I38T + S60P and I38T + I201T were tested in this study (Figure S2). However, these substitutions were unable to restore the growth impairment of the I38T‐substituted viruses. Given that I38‐substituted viruses show different patterns in terms of replicative capacity dependent on the type/subtype or isolated year,^11^, ^19^, ^26^, ^28^ different substitutions are likely to be involved in restoration of the replicative capacity of the I38 mutant viruses. In addition, currently circulating strains may have a different genetic background compared with the recombinant viruses used in this study. While in vitro results of the replicative capacity of I38X mutant viruses from different studies vary, further investigation of potential compensatory mutations that could recover the fitness cost of I38T substitution will be needed.

It is important for disease management to understand the risk of treatment‐emergent resistance to BXA, and therefore, continuous surveillance and exploration of mutations that affect BXA susceptibility and viral fitness are important. This study provides the characteristics of clinically identified I38‐substituted viruses, and extensive information on the impacts of further non‐I38 AA substitutions in the trimeric viral polymerase complex to BXA susceptibility. This additional information will further support influenza surveillance.

## CONFLICT OF INTEREST

TH, KB, KI, MO, TN, SH, TS, AN, and SO are employed by Shionogi and Co., Ltd. SW is employed by F. Hoffmann‐La Roche Ltd.

## AUTHOR CONTRIBUTIONS

**Takashi Hashimoto:** Conceptualization (equal); Data curation (equal); Formal analysis (lead); Writing‐original draft (lead); Writing‐review & editing (equal). **Keiko Baba:** Conceptualization (supporting); Data curation (supporting); Formal analysis (supporting); Writing‐review & editing (supporting). **Kae Inoue:** Data curation (supporting); Formal analysis (supporting). **Miyako Okane:** Data curation (supporting); Formal analysis (supporting). **Satoshi Hata:** Conceptualization (supporting); Funding acquisition (supporting); Resources (supporting); Supervision (supporting). **Takao Shishido:** Conceptualization (equal); Data curation (equal); Funding acquisition (equal); Resources (lead); Supervision (equal); Writing‐review & editing (equal). **Akira Naito:** Conceptualization (equal); Data curation (lead); Funding acquisition (lead); Resources (equal); Supervision (equal); Writing‐review & editing (equal). **Steffen Wildum:** Conceptualization (lead); Data curation (equal); Funding acquisition (lead); Supervision (lead); Writing‐original draft (equal); Writing‐review & editing (lead). **Shinya Omoto:** Conceptualization (lead); Data curation (equal); Funding acquisition (supporting); Supervision (lead); Writing‐original draft (equal); Writing‐review & editing (lead).

### PEER REVIEW

The peer review history for this article is available at https://publons.com/publon/10.1111/irv.12821.

## Supporting information

Supplementary Material

## Data Availability

All relevant data are within the manuscript and its Supporting Information.
