# Supplementary material for: Comprehensive assessment of amino acid substitutions in the trimeric RNA polymerase complex of influenza A virus detected in clinical trials of baloxavir marboxil
Source: Influenza Other Respir Viruses. 2020 Oct 24;15(3):389–95. doi: 10.1111/irv.12821 (PMC8051730; doi:10.1111/irv.12821)

**Supplementary Table 1. Drug susceptibility of recombinant viruses with amino acid substitutions to baloxavir and favipiravir in the plaque reduction assay.**

.

|  |  | **BXA** | | |  | **Favipiravir** | | |
| --- | --- | --- | --- | --- | --- | --- | --- | --- |
|  |  | **EC_50_ (nmol/L)** | | **FC** |  | **EC_50_ (nmol/L)** | | **FC** |
| **Strains** | **Clinical study** | **Mean** | **SD** |  |  | **Mean** | **SD** |  |
| ***Experiment 1*** |  |  |  |  |  |  |  |  |
| rgA/WSN/33 (H1N1) | N/A | 0.42 | 0.12 | **N/A** |  | 17382.98 | 9432.24 | **N/A** |
| rgA/WSN/33-PB2/A221T | T0821 | 0.38 | 0.06 | **0.90** |  | 21485.12 | 13573.49 | **1.24** |
| rgA/WSN/33-PB2/I310M | T0821 | 0.29 | 0.08 | **0.71** |  | 13135.98 | 1826.43 | **0.76** |
| rgA/WSN/33-PB2/T333I | T0821 | 0.24 | 0.02 | **0.58** |  | 13159.46 | 1392.14 | **0.76** |
| rgA/WSN/33-PB1/M92T | T0821 | 0.33 | 0.05 | **0.79** |  | 28677.10 | 12437.15 | **1.65** |
| rgA/WSN/33-PB1/V418I | T0821 | 0.30 | 0.10 | **0.71** |  | 13022.09 | 4187.71 | **0.75** |
| ***Experiment 2*** |  |  |  |  |  |  |  |  |
| rgA/Victoria/3/75 (H3N2) | N/A | 1.15 | 0.59 | **N/A** |  | 9391.13 | 695.68 | **N/A** |
| rgA/Victoria/3/75-PB1/I205M | T0822 | 0.73 | 0.17 | **0.63** |  | 8880.34 | 4501.72 | **0.95** |
| rgA/Victoria/3/75-PB1/G250E | T0822 | Not rescued | | | | | | |
| rgA/Victoria/3/75-PA/I38T+PB1/G250E | T0822 | Not rescued | | | | | | |
| rgA/Victoria/3/75-PB1/M290T | T0822 | 0.39 | 0.24 | **0.34** |  | 3075.20 | 1280.24 | **0.33** |
| rgA/Victoria/3/75-PB2/D60G | T0822 | 1.06 | 0.15 | **0.92** |  | 5353.79 | 1910.41 | **0.57** |
| rgA/Victoria/3/75-PA/I38T+PB2/D60G | T0822 | 49.37 | 19.05 | **42.85** |  | 2941.86 | 3.96 | **0.31** |
| rgA/Victoria/3/75-PB2/V105M | T0822 | 0.67 | 0.18 | **0.58** |  | 4414.58 | 336.40 | **0.47** |
| rgA/Victoria/3/75-PB2/E171K | T0822 | Not rescued | | | | | | |
| rgA/Victoria/3/75-PB2/K197R | T0822 | 1.56 | 0.69 | **1.36** |  | 10184.53 | 3765.78 | **1.08** |
| rgA/Victoria/3/75-PA/I38T+PB2/K197R | T0822 | 23.12 | 20.73 | **20.07** |  | 2260.12 | 549.91 | **0.24** |
| rgA/Victoria/3/75-PB2/K353R | T0822 | 0.84 | 0.22 | **0.73** |  | 5762.89 | 2278.97 | **0.61** |
| rgA/Victoria/3/75-PB2/I385V | T0822 | 0.74 | 0.13 | **0.64** |  | 5777.87 | 176.93 | **0.62** |
| rgA/Victoria/3/75-PA/I38V | Not clinically detected  Reference^10^ | 2.11 | 0.81 | **1.83** |  | 11013.30 | 2541.61 | **1.17** |
| rgA/Victoria/3/75-PA/E23G | T0831 | 2.75 | 1.48 | **2.39** |  | 3181.57 | 615.22 | **0.34** |
| rgA/Victoria/3/75-PA/G99E | T0831 | 0.71 | 0.28 | **0.61** |  | 4467.93 | 1815.40 | **0.48** |
| rgA/Victoria/3/75-PA/A183V | T0831 | 0.59 | 0.40 | **0.51** |  | 6773.81 | 383.25 | **0.72** |
| rgA/Victoria/3/75-PA/G186D | T0831 | 0.21 | 0.13 | **0.18** |  | 5190.20 | 3088.27 | **0.55** |
| rgA/Victoria/3/75-PA/I201T | T0831 | 1.26 | 0.61 | **1.10** |  | 7489.31 | 1044.17 | **0.80** |
| rgA/Victoria/3/75-PA/I38T+I201T | T0831 | 39.09 | 5.29 | **33.92** |  | 1932.55 | 335.66 | **0.21** |
| rgA/Victoria/3/75-PA/R212C | T0831 | 0.79 | 0.33 | **0.68** |  | 6501.19 | 2074.66 | **0.69** |
| rgA/Victoria/3/75-PA/S224F | T0831 | 0.90 | 0.84 | **0.78** |  | 4563.87 | 1945.03 | **0.49** |
| rgA/Victoria/3/75-PA/A231V | T0831 | 0.67 | 0.30 | **0.58** |  | 8001.15 | 1271.80 | **0.85** |
| rgA/Victoria/3/75-PA/C241F | T0831 | 0.65 | 0.17 | **0.56** |  | 4978.25 | 2860.02 | **0.53** |
| rgA/Victoria/3/75-PA/E23G+C241F | T0831 | 2.04 | 1.35 | **1.77** |  | 3158.62 | 855.65 | **0.34** |
| rgA/Victoria/3/75-PA/P271S | T0831 | 0.60 | 0.22 | **0.52** |  | 7105.85 | 1839.72 | **0.76** |
| rgA/Victoria/3/75-PA/G299R | T0831 | 1.54 | 0.77 | **1.34** |  | 10511.64 | 1548.37 | **1.12** |
| rgA/Victoria/3/75-PA/G316R | T0831 | 0.30 | 0.07 | **0.26** |  | 4379.94 | 4135.61 | **0.47** |
| rgA/Victoria/3/75-PA/T357A | T0831 | 1.07 | 0.86 | **0.93** |  | 5553.64 | 2464.55 | **0.59** |
| rgA/Victoria/3/75-PA/R385K | T0831 | 1.22 | 0.46 | **1.06** |  | 8492.67 | 1279.21 | **0.90** |
| rgA/Victoria/3/75-PA/S395N | T0831 | 0.70 | 0.44 | **0.60** |  | 4855.33 | 3598.11 | **0.52** |
| rgA/Victoria/3/75-PA/S405C | T0831 | 0.80 | 0.62 | **0.69** |  | 9113.79 | 2301.91 | **0.97** |
| rgA/Victoria/3/75-PA/I421T | T0831 | 1.26 | 1.14 | **1.10** |  | 9011.35 | 2025.41 | **0.96** |
| rgA/Victoria/3/75-PA/L482I | T0831 | 0.60 | 0.06 | **0.52** |  | 6417.94 | 2100.63 | **0.68** |
| rgA/Victoria/3/75-PA/E493G | T0831 | 0.51 | 0.39 | **0.44** |  | 3352.08 | 557.85 | **0.36** |
| rgA/Victoria/3/75-PA/I545M | T0831 | 0.49 | 0.17 | **0.43** |  | 5008.19 | 1600.33 | **0.53** |
| rgA/Victoria/3/75-PA/M561I | T0831 | 1.05 | 0.23 | **0.91** |  | 12651.59 | 2690.36 | **1.35** |
| rgA/Victoria/3/75-PA/V602I | T0831 | 1.31 | 0.76 | **1.14** |  | 7293.76 | 837.96 | **0.78** |
| rgA/Victoria/3/75-PA/E623G | T0831 | 1.20 | 0.72 | **1.04** |  | 11898.38 | 2639.52 | **1.27** |
| rgA/Victoria/3/75-PA/E630K | T0831 | 0.46 | 0.19 | **0.40** |  | 5716.38 | 1305.31 | **0.61** |
| rgA/Victoria/3/75-PA/G316R+E630K | T0831 | 0.41 | 0.18 | **0.36** |  | 4811.41 | 2474.79 | **0.51** |
| rgA/Victoria/3/75-PA/L649M | T0831 | 0.47 | 0.10 | **0.41** |  | 4391.30 | 2592.85 | **0.47** |
| rgA/Victoria/3/75-PA/V668I | T0831 | 0.93 | 0.48 | **0.81** |  | 6218.19 | 1961.65 | **0.66** |
| rgA/WSN/33 (H1N1) | N/A | 0.45 | 0.22 | **N/A** |  | 14991.68 | 1395.47 | **N/A** |
| rgA/WSN/33-PA/I38V | Reference^10^ | 0.97 | 0.80 | **2.18** |  | 13027.98 | 2249.52 | **0.87** |
| rgB/Maryland/1/59 | N/A | 10.07 | 5.45 | **N/A** |  | 28482.49 | 13300.11 | **N/A** |
| rgB/Maryland/1/59-PA/T60V | T0831 | 8.63 | 3.28 | **0.86** |  | 38146.56 | 11888.18 | **1.34** |
| rgB/Maryland/1/59-PA/D112N | T0831 | 6.17 | 3.22 | **0.61** |  | 35759.07 | 14604.90 | **1.26** |
| rgB/Maryland/1/59-PA/E333K | T0831 | 7.08 | 1.88 | **0.70** |  | 28127.17 | 8846.82 | **0.99** |
| rgB/Maryland/1/59-PA/Y361H | T0831 | 10.42 | 3.70 | **1.03** |  | 38213.73 | 7199.86 | **1.34** |
| ***Experiment 3*** |  |  |  |  |  |  |  |  |
| rgA/Victoria/3/75 (H3N2) | N/A | 1.05 | 0.35 | **N/A** |  | 7060.57 | 2233.88 | **N/A** |
| rgA/Victoria/3/75-PB1/A231V | T0831 | Not rescued | | | | | | |
| rgA/Victoria/3/75-PB1/I517M | T0831 | 1.02 | 0.18 | **0.97** |  | 6151.12 | 1429.04 | **0.87** |
| rgA/Victoria/3/75-PB2/R101G | T0831 | 0.85 | 0.14 | **0.80** |  | 7822.63 | 3584.65 | **1.11** |
| rgA/Victoria/3/75-PB2/M202L | T0831 | 1.80 | 0.36 | **1.70** |  | 8300.60 | 1613.03 | **1.18** |
| rgA/Victoria/3/75-PB2/R209K | T0831 | 0.55 | 0.15 | **0.53** |  | 5811.18 | 2497.40 | **0.82** |
| rgA/Victoria/3/75-PB2/M475I | T0831 | 1.38 | 0.37 | **1.31** |  | 9414.59 | 1102.43 | **1.33** |
| rgA/Victoria/3/75-PB2/P585L | T0831 | Not rescued | | | | | | |
| rgA/Victoria/3/75-PB2/M475I+P585L | T0831 | Not rescued | | | | | | |
| ***Experiment 4*** |  |  |  |  |  |  |  |  |
| rgA/WSN/33 (H1N1) | N/A | 0.36 | 0.03 | **N/A** |  | 29671.00 | 12911.66 | **N/A** |
| rgA/WSN/33-PA/I38N | T0832 | 8.52 | 2.87 | **23.66** |  | 8334.63 | 1785.08 | **0.28** |
| rgA/WSN/33-PA/E397G | T0832 | 0.33 | 0.08 | **0.92** |  | 35457.66 | 1163.52 | **1.20** |
| rgA/Victoria/3/75 (H3N2) | N/A | 0.73 | 0.41 | **N/A** |  | 5702.37 | 2812.94 | **N/A** |
| rgA/Victoria/3/75-PA/L28V | T0832 | 1.47 | 0.78 | **2.02** |  | 6076.36 | 2790.96 | **1.07** |
| rgA/Victoria/3/75-PA/K34E | T0832 | 1.43 | 1.60 | **1.96** |  | 3434.02 | 1704.84 | **0.60** |
| rgA/Victoria/3/75-PA/P68L | T0832 | 0.89 | 0.40 | **1.23** |  | 6495.30 | 258.72 | **1.14** |
| rgA/Victoria/3/75-PA/L71M | T0832 | 0.46 | 0.08 | **0.64** |  | 5301.43 | 916.52 | **0.93** |
| rgA/Victoria/3/75-PA/V90A | T0832 | 0.83 | 0.45 | **1.14** |  | 4994.87 | 653.58 | **0.88** |
| rgA/Victoria/3/75-PA/T98N | T0832 | 0.38 | 0.08 | **0.52** |  | 7210.91 | 1806.28 | **1.26** |
| rgA/Victoria/3/75-PA/D160G | T0832 | 0.59 | 0.16 | **0.81** |  | 5826.92 | 3045.04 | **1.02** |
| rgA/Victoria/3/75-PA/F191L | T0832 | Not rescued | | | | | | |
| rgA/Victoria/3/75-PA/R192H | T0832 | 0.62 | 0.10 | **0.85** |  | 4647.34 | 1183.45 | **0.81** |
| rgA/Victoria/3/75-PA/E397K | T0832 | 0.56 | 0.39 | **0.77** |  | 5760.20 | 711.64 | **1.01** |
| rgB/Maryland/1/59 | N/A | 7.60 | 5.22 | **N/A** |  | 24566.71 | 11313.42 | **N/A** |
| rgB/Maryland/1/59-PA/R7K | T0832 | 9.45 | 3.43 | **1.24** |  | 28662.78 | 10965.92 | **1.17** |
| rgB/Maryland/1/59-PA/S25G | T0832 | 7.20 | 1.80 | **0.95** |  | 25411.38 | 12480.92 | **1.03** |
| rgB/Maryland/1/59-PA/T62K | T0832 | 3.67 | 1.25 | **0.48** |  | 26733.39 | 12212.48 | **1.09** |
| rgB/Maryland/1/59-PA/D201E | T0832 | 7.58 | 1.89 | **1.00** |  | 26496.40 | 13694.44 | **1.08** |
| rgB/Maryland/1/59-PA/D201G | T0832 | 10.32 | 1.08 | **1.36** |  | 30726.50 | 11772.75 | **1.25** |
| rgB/Maryland/1/59-PA/E333G | T0832 | 9.58 | 2.70 | **1.26** |  | 26014.45 | 12568.62 | **1.06** |
| rgB/Maryland/1/59-PA/S415G | T0832 | 9.30 | 2.45 | **1.22** |  | 25276.62 | 14820.06 | **1.03** |
| rgB/Maryland/1/59-PA/S415N | T0832 | 11.91 | 0.72 | **1.57** |  | 30915.94 | 12243.84 | **1.26** |
| ***Experiment 5*** |  |  |  |  |  |  |  |  |
| rgA/WSN/33 (H1N1) | N/A | 0.64 | 0.35 | **N/A** |  | 13725.93 | 3450.67 | **N/A** |
| rgA/WSN/33-PA/I38S | Reference^18,26^ | 7.90 | 3.90 | **12.43** |  | 8936.67 | 1466.88 | **0.65** |
| rgA/WSN/33-PA/I38R | T0832 | Not rescued | | | | | | |
| rgA/WSN/33-PA/I38L | Reference^25^ | 4.02 | 3.51 | **6.33** |  | 24471.45 | 11211.86 | **1.78** |
| rgA/Victoria/3/75 (H3N2) | N/A | 1.65 | 0.69 | **N/A** |  | 7264.94 | 6919.86 | **N/A** |
| rgA/Victoria/3/75-PA/I38N | Not clinically detected | 17.01 | 9.37 | **10.32** |  | 5229.60 | 3574.09 | **0.72** |
| rgA/Victoria/3/75-PA/I38S | Not clinically detected | 9.63 | 5.93 | **5.85** |  | 3948.28 | 3796.63 | **0.54** |
| rgA/Victoria/3/75-PA/I38R | T0832 | Not rescued | | | | | | |
| rgA/Victoria/3/75-PA/I38L | Not clinically detected | 3.57 | 2.51 | **2.17** |  | 8839.97 | 1501.91 | **1.22** |

EC_50_ of baloxavir acid and favipiravir were determined by plaque reduction assay. Data represent the mean and standard deviation (SD) of more than three times of independent experiments. Fold change (FC) was calculated as relative EC_50_ of each tested virus to that of the cognate wild-type virus. N/A, not applicable.

**Supplementary Table 2. Drug susceptibility of recombinant viruses with amino acid substitutions to baloxavir and favipiravir in the ViraDot Assay.**

| **Strains** | **Rationale for evaluation** |  | **BXA** | | |  | **Favipiravir** | | |
| --- | --- | --- | --- | --- | --- | --- | --- | --- | --- |
|  |  |  | **EC_50_ (nmol/L)** | | **FC** |  | **EC_50_ (μmol/L)** | | **FC** |
|  |  |  | **Mean** | **SD** |  |  | **Mean** | **SD** |  |
| rgA/WSN/33 (H1N1) | Reference / Assay control |  | 0.28 | 0.03 | **N/A** |  | 63.3 | 10.4 | **N/A** |
| rgA/WSN/33-PA/I38T |  |  | 7.52 | 0.63 | **26.58** |  | 47.5 | 11.5 | **0.74** |
| rgA/Victoria/3/75 (H3N2) |  |  | 0.42 | 0.05 | **N/A** |  | 81.3 | 28.5 | **N/A** |
| rgA/Victoria/3/75-PA/E23G |  |  | 1.41 | 0.36 | **3.37** |  | 72.1 | 24.5 | **0.89** |
| rgA/Victoria/3/75-PA/I38T |  |  | 11.12 | 4.03 | **26.39** |  | 73.5 | 24.7 | **0.90** |
| rgB/Maryland/1/59 |  |  | 3.13 | 0.39 | **N/A** |  | 18.0 | 5.5 | **N/A** |
| rgB/Maryland/1/59-PA/I38T |  |  | 47.24 | 8.34 | **15.02** |  | 15.7 | 4.6 | **0.88** |
| rgA/WSN/33-PA/A37S | Substitutions identified at amino acid positions associated with resistance in NCBI  database sequences. |  | 0.41 | 0.08 | **1.27** |  | 78.6 | 11.9 | **0.98** |
| rgA/WSN/33-PA/E199D |  |  | 0.43 | 0.07 | **1.35** |  | 79.6 | 11.4 | **0.99** |
| rgA/Victoria/3/75-PA/E199D |  |  | 0.49 | 0.23 | **1.33** |  | 124.1 | 14.6 | **1.24** |
| rgA/Victoria/3/75-PA/V62I | Baseline substitutions associated with reduced virologic response. |  | 0.39 | 0.12 | **1.09** |  | 108.7 | 12.9 | **1.09** |
| rgA/Victoria/3/75-PA/K492R |  |  | 0.40 | 0.16 | **1.10** |  | 115.1 | 8.8 | **1.15** |
| rgB/Maryland/1/59-PA/M682L |  |  | 3.46 | 0.30 | **1.21** |  | 32.1 | 3.0 | **0.86** |
| rgA/Victoria/3/75-PB2/R209K | Treatment-emergent substitutions associated with virologic  rebound. |  | 0.64 | 0.10 | **0.88** |  | 130.7 | 37.1 | **0.61** |
| rgA/WSN/33-PA/P267S | Polymorphisms associated with elevated (≥90 percentile)  baseline EC_50_ values of virus isolated from clinical specimens  within trials. |  | 0.46 | 0.06 | **1.47** |  | 85.3 | 15.2 | **1.06** |
| rgA/WSN/33-PA/A476S |  |  | 0.46 | 0.07 | **1.45** |  | 76.9 | 9.8 | **0.96** |
| rgA/WSN/33-PA/E677D |  |  | 0.30 | 0.04 | **1.06** |  | 81.9 | 6.6 | **0.78** |
| rgA/Victoria/3/75-PA/F35L |  |  | 0.35 | 0.07 | **1.02** |  | 109.0 | 9.0 | **1.09** |
| rgA/Victoria/3/75-PA/T162I |  |  | 0.63 | 0.11 | **1.13** |  | 121.3 | 26.5 | **1.13** |
| rgA/Victoria/3/75-PA/Y321H |  |  | 0.58 | 0.06 | **1.06** |  | 107.1 | 17.8 | **1.00** |
| rgA/Victoria/3/75-PA/V432I |  |  | 0.78 | 0.03 | **1.01** |  | 183.2 | 24.9 | **0.89** |
| rgA/Victoria/3/75-PA/M595I |  |  | 0.57 | 0.14 | **1.02** |  | 128.0 | 24.6 | **1.20** |
| rgA/Victoria/3/75-PA/A618S |  |  | 0.62 | 0.18 | **1.08** |  | 100.4 | 25.2 | **0.93** |
| rgA/Victoria/3/75-PA/G684R |  |  | 0.52 | 0.03 | **0.95** |  | 95.9 | 29.6 | **0.89** |
| rgB/Maryland/1/59-PA/G199R |  |  | 2.44 | 0.44 | **0.84** |  | 34.1 | 3.1 | **0.91** |
| rgB/Maryland/1/59-PA/K298R |  |  | 3.28 | 0.59 | **1.13** |  | 38.8 | 2.1 | **1.04** |
| rgB/Maryland/1/59-PA/T304A |  |  | 3.68 | 0.45 | **1.28** |  | 39.6 | 1.4 | **1.06** |
| rgB/Maryland/1/59-PA/V645A |  |  | 3.50 | 0.35 | **1.22** |  | 26.0 | 3.9 | **0.70** |

EC_50_ of baloxavir acid and favipiravir were determined using the ViraDot assay. Data represent the mean and standard deviation (SD) of three independent experiments. Fold change (FC) was calculated as relative EC_50_ of each tested virus to that of the cognate wild-type virus. N/A, not applicable; NCBI, National Center for Biotechnology Information.

**Supplementary Figure 1.** **Replicative capacity of variant viruses with indicated PA/I38 substitutions in RPMI2650 cells.** Human RPMI2650 cells were infected with wild-type (WT) or indicated mutant viruses based on rgA/WSN/33 (H1N1) (A, C), or rgA/Victoria/3/75 (H3N2) (B, D). The culture supernatants were collected at the indicated time points and viral titers (TCID_50_/mL) were determined in MDCK cells. Each plot represents the mean and standard deviation of triplicate experiments. The lower limit of quantification of the virus titers was indicated by a dashed line. **P* < 0.05 to WT by Welch’s *t*-test at the indicated time points.


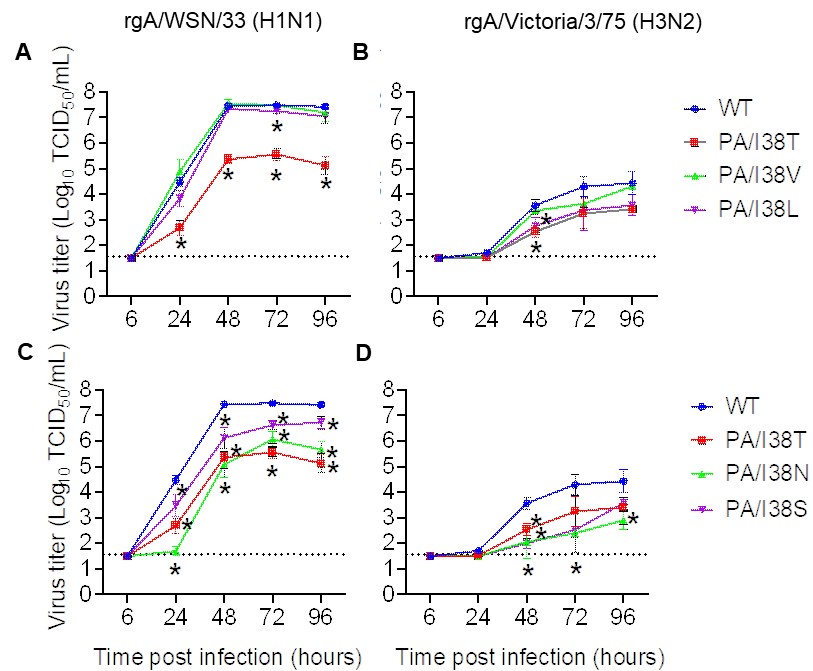


**Supplementary Figure 2.** **Replicative capacity of variant viruses with indicated amino acid substitutions in PA protein in MDCK cells and RPMI2650 cells**

MDCK cells (A, C) or RPMI2650 cells (B, D) were infected with wild-type (WT) or amino acid-substituted viruses based on rgA/Victoria/3/75 (H3N2). The culture supernatants were collected at the indicated time points and viral titers (TCID_50_/mL) were determined in MDCK cells. Each symbol represents the mean and standard deviation of triplicate experiments. The lower limit of quantification of the virus titers was indicated by a dashed line.


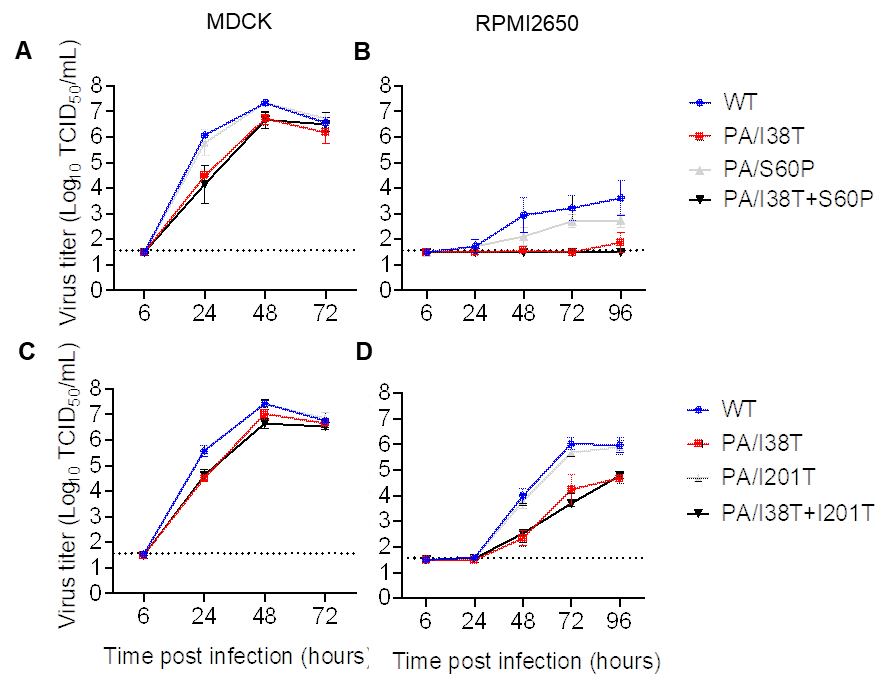

Supplement: Supplementary file 1 — Supplementary Material [file IRV-15-389-s001.docx]
